# Numerical investigation of ibuprofen removal from pharmaceutical wastewater using adsorption process

**DOI:** 10.1038/s41598-021-04185-9

**Published:** 2021-12-29

**Authors:** Yan Cao, Ali Taghvaie Nakhjiri, Mahdi Ghadiri

**Affiliations:** 1 School of Computer Science and Engineering, Xi’an Technological University, Xi’an, 710021 China; 2 Department of Petroleum and Chemical Engineering, Science and Research Branch, Islamic Azad University, Tehran, Iran; 3 Department of Chemical Sciences, Bernal Institute, University of Limerick, Limerick, Ireland

**Keywords:** Chemical engineering, Chemical biology, Medicinal chemistry, Pharmaceutics

## Abstract

In the present study, a mathematical modelling was developed to investigate ibuprofen adsorption from pharmaceutical wastewater into activated carbon and sonicated activated carbon. The developed model was dissolved based on the finite element method. Effect of different operating parameters including particle porosity and diameter as well as ibuprofen diffusion coefficient in solution on the amount of ibuprofen adsorption at different time point and position in the particle were evaluated. It was found good agreement between experimental values and modelling results in terms of ibuprofen adsorption as a function time. The 84.5% and 92.5% of maximum adsorption was achieved for the AC and SAC at the centre of particle after 150 min. Increasing the particle porosity and ibuprofen diffusion coefficient was improved the ibuprofen adsorption into the adsorbent. However, the particle diameter had negative impact on the system performance. There was a decrease in solute adsorption from 84.10 to 7.30 mg/g and from 106 to 15.73 mg/g for the AC and SAC respectively with increasing the particle radius from 173 to 500 µm. Finally, it was concluded that the particle specifications play important role in the adsorption process as it was observed considerable change in the amount of adsorption at different positions in the particle with changing the particle specifications.

## Introduction

Adsorption of a solute in a liquid on a solid is one of the most important processes in science and engineering. It consists of three steps including diffusion of the solute from bulk liquid phase on the external surface of solid, diffusion of adsorbed solute into the pores of solid, and adsorption of the solute on the active sites of adsorbent^1,2^. The industrial wastewaters and effluents are considered any streams containing any solute which are undesirable and increase the pollution of environment^3^. It is highly important to deal with these wastewaters in order to prevent soil, air, and groundwater pollution. Electro-Fenton process was used to remove metformin and mefenamic acid from pharmaceutical wastewater^4,5^. Adsorption process has been used widely for removal of heavy metals^6^, pharmaceutical contaminants^7,8^, the removal of dyes^9^. Pharmaceutical contaminants can cause significant negative impact on humans and animals, even at tiny amount^10–12^. Ibuprofen is one of the active pharmaceutical ingredients which has been found at concentrations of up to mg/L in many pharmaceutical wastewaters. It is a non-steroidal, anti-inflammatory, colourless, crystalline solid with a characteristic odour. It is usually used for the treatment of fever, muscle pain and inflammation^10^. Stewart Adams discovered Ibuprofen 1961 and it has been used several areas for the treatment of diseases^7^.

Many researchers have been used adsorption process as a reliable approach as well as its advantages including low cost, simple setup and operation to remove ibuprofen from pharmaceutical wastewater^13^. Kollarahithlu and Balakrishnan^14^ used amine functionalized superparamagnetic silica nanocomposite for ibuprofen removal and it was achieved 97% removal within the first 15 min. Zeolite-rich composites was used for the treatment of wastewater containing ibuprofen and the adsorbent capacity was obtained 19.7 mg/g for ibuprofen. Also, presence of inorganic anions such as sulphates and bicarbonates was decreased the efficiency of bilayer composites^15^. Adsorption of ibuprofen on cocoa shell biomass-based adsorbents was investigated and it was concluded that the ibuprofen adsorption is endothermic and π–π stacking and van der Waals interactions were involved in its adsorption on the solid^16^. Tetracycline antibiotic adsorption onto modified zeolite was investigated experimentally and theoretically by Dolatabadi et al.^17^.

Different kinetic models such as Langmuir and Freundlich models are used for the investigation of the process kinetic. Martin et al.^18^ investigated Ibuprofen adsorption modified mica and montmorillonite and it was proposed that Ibuprofen adsorption onto modified mica can be described by Freundlich model while Langmuir model is suitable for montmorillonite. Furthermore, a three-dimensional mass transfer model was proposed for the investigation of ibuprofen adsorption on an activated carbon^19^. Based on the developed model results, total intraparticular flux was a function of time as well as position inside the particle. Also, it was found that surface diffusion plays major role for the diffusion of the ibuprofen molecule and diffusion in the pore volume is ignorable^19^. A model was developed for the prediction of the composite equilibrium isotherm in cadmium ions adsorption onto nanochitosan^20^. A number of simulation has been performed in packed bed scale^21,22^. Reynolds mass flux model was used for the simulation of methylene chloride adsorption on activated carbon in a packed column. It was able to determine different things in the system such as mass and heat transfer, the system hydrodynamic, and breakthrough/regeneration curves^21,22^. Moreover, a mathematical modelling was developed for the evaluation of acetaminophen removal from pharmaceutical wastewater in a fixed-bed adsorption column using sugarcane bagasse^23^. It was found that increase in bed height led to the enhancement of mass transfer zone and in the system Knudsen diffusion was considerably higher than the pore diffusion^23^. As it can be seen that there is no study at particle scale in order to investigation of the particle characteristic on the solute adsorption on an absorbent. Therefore, it is highly important to provide a proper model for evaluation of the process for a one system and even it can be used for other systems with different solute and adsorbent.

In the current study, an unsteady mathematical model was developed in order to evaluate adsorption of ibuprofen on activated carbon (AC) and sonicated activated carbon (SAC). The evaluation of particle specification and solute properties on ibuprofen adsorption at different time and in different position of particle is the novelty of the current study. The COMSOL Multiphysics was used for the development of mathematical model. The developed model was validated with experimental data. The effect of operating parameters including the particle diameter, mass transfer coefficient, porosity and density of particle, on the amount of ibuprofen adsorption was investigated.

## Model development

Adsorption of ibuprofen on a sonicated activated carbon was selected as a case study for the development of a mathematical model. Molecular diffusion model was used in this study. This model considers external resistance to mass transfer. Using local diffusion model has a number of advantages in comparison with global model. It is because the effective diffusion coefficient is only function of particle in the local diffusion model. The model is based on the following assumption:

1. Equilibrium between fluid and solid is instantaneously reached
2. Surface diffusion is predominant
3. Solid phase was considered as a homogeneous medium.
4. Isothermal process, perfect mixing, spherical particles, and single component

The system was also assumed to be isotherm. In global models, the system parameters are function of variables such as fluid velocity and the particle diameter^24–26^.

1 $$\frac{\partial q}{{\partial t}} = \frac{1}{{r^{2} }}\frac{\partial }{\partial r}\left( {D_{eff} r^{2} \frac{\partial q}{{\partial r}}} \right),$$

where q is the amount of solute retained in the solid phase (mg/g) and *D*_*eff*_ is effective diffusion coefficient (m^2^/s). Two boundary conditions and an initial condition are required for solving the Eq. (1)^24^.

2 $$q = 0\quad t = 0,$$

3 $$- D_{eff} \rho_{p} \frac{\partial q}{{\partial r}} = k_{m} \varepsilon \rho_{l} (C_{eq} - C),\,\,\,\,\,r = \frac{{d_{p} }}{2},$$

4 $$\frac{\partial q}{{\partial r}} = 0,\,\,\,\,\,\,\,\,\,r = 0,$$

where ρ_P_, C_eq_, and k_m_ are the particle density, the solute concentration at equilibrium state, and mass transfer coefficient of external mass transfer. Sherwood equation is used for the determination of mass transfer coefficient.

Langmuir model isotherm was used to convert the concentration (C) to the amount of adsorption (q) in order to present equilibrium results. There is equilibrium between ibuprofen in solution and ibuprofen in solid^24^.

5 $$q = \frac{{q_{m} bC}}{1 + bC},$$

where C (mg/L), b (L/mg) and *q*_*m*_ (mg/g) are the equilibrium concentration in the solution, the Langmuir adsorption equilibrium constant, and the maximum adsorption capacity respectively. The maximum adsorption capacity is obtained using experimental work.

The Eq. (5) was written in the form of concentration as a function of amount of adsorption and was replaced in the Eq. (3) in order to solve Eq. (1). The composite equation can be written as Eq. (8)^24^.

6 $$C=\frac{q}{b{q}_{m}-bq},$$

7 $$\frac{\partial q}{\partial r}=-\frac{{k}_{m\varepsilon {\rho }_{l}}}{{D}_{eff}{\rho }_{p}}\left({C}_{eq}-\frac{q}{b{q}_{m}-bq}\right) r=\frac{{d}_{p}}{2},$$

8 $$\frac{\partial q}{\partial t}=-\frac{1}{{r}^{2}}\frac{\partial }{\partial r}\left({r}^{2}.\frac{{k}_{m\varepsilon {\rho }_{l}}}{{\rho }_{p}}\left({C}_{eq}-\frac{q}{b{q}_{m}-bq}\right)\right).$$

The Eq. (1) was solved with appropriate boundary conditions using COMSOL Multiphysics software. Brilliant positive points such as robustness and flexibility of solving disparate types of stiff/non-stiff boundary problems have motivated the investigators to employ this efficacious software^27–29^. The developed model was solved in one-dimensional. It is because the particles are spherical and displacement on the surface of particle does not make any changes related to $\theta$ and $\varphi$ coordinates. The Eq. (1) in spherical coordinates can be written as follows^24^:

9 $$\begin{aligned} \frac{\partial q}{{\partial t}} & - D_{eff} \left[ {\frac{1}{{r^{2} }}\frac{\partial }{\partial r}\left( {r^{2} \frac{\partial q}{{\partial r}}} \right) + \frac{1}{{r^{2} \sin \theta }}\frac{\partial }{\partial \theta }\left( {\sin \theta \frac{\partial q}{{\partial \theta }}} \right)} \right. \\ & \left. { + \frac{1}{{r^{2} \sin^{2} \theta }}\frac{{\partial^{2} q}}{{\partial \varphi^{2} }}} \right] = 0. \\ \end{aligned}$$

It was assumed that the all particles are same size and amount adsorption in $\theta$ and $\varphi$ directions are zero^24^.

10 $$\frac{\partial q}{{\partial \theta }} = 0,$$

11 $$\frac{\partial q}{{\partial \varphi }} = 0.$$

Therefore, the Eq. (9) can be expressed like Eq. (1). The whole equation was multiplied by r^2^ in order to prevent creation of problem in equation solving at r = 0^24^.

12 $$r^{2} \frac{\partial q}{{\partial t}} + \frac{\partial }{\partial r}\left( { - D_{eff} r^{2} \frac{\partial q}{{\partial r}}} \right) = 0.$$

Moreover, it can be used a dimensionless radial coordinate to facilitate the equation solving. Also, it was not required to change the geometry in the simulation by using dimensionless radial coordinate^24^.

13 $$\frac{\partial }{\partial r}=\frac{1}{R}\frac{\partial }{\partial \widehat{r}},$$

14 $${\widehat{r}}^{2}\frac{\partial q}{\partial t}+\frac{\partial }{\partial \widehat{r}}\left(-\frac{{D}_{eff}{\widehat{r}}^{2}}{{R}^{2}}\frac{\partial q}{\partial \widehat{r}}\right)=0.$$

Therefore, the Eq. (14) was defined in the software. The R is the particle radius. Two Neumann boundary conditions and one initial condition was required to solve the equation. The flux is zero at centre due to existence of symmetrical geometry^24^.

15 $$\frac{\partial q}{{\partial \hat{r}}} = 0.$$

On the particle surface ($\hat{r} = 1$) the equation can be written as follows^24^:

16 $$- D_{eff} \rho_{p} \frac{\partial q}{{\partial r}} = k_{m} \varepsilon \rho_{l} (C_{eq} - C),\,\,\,\,\,r = \frac{{d_{p} }}{2}.$$

The developed model is used for the evaluation of ibuprofen adsorption on the activated carbon. The operating parameters used in the developed model were provided in Table 1.

**Table 1:** Particle characteristic and operating conditions^19,30–32^.

Symbol	Parameter	Value	Unit
ρ_solution_	Solution density	1000	kg/m^3^
ρ_solid_	Solid density	2110	kg/m^3^
D_AB_	Diffusion coefficient in solution	(1–10)e^−10^	m^2^/s
R_particel_ (R)	Radius of particle	173–500	µm
ε	Particle porosity	0.3–0.7	–
k_m_	External mass transfer coefficient	(1–1.4)10^–4^	m/s
q_m_	Maximum capacity	84.9 or 106	mg/g
S_BET-AC_	AC BET surface area	641	m^2^/g
S_BET-SAC_	SAC BET surface area	732	m^2^/g
PD_AC_	AC average pore diameter (PD)	3.88	nm
PD_AC_	SAC average pore diameter (PD)	4.15	nm

## Results and discussion

### Model validation

**Figure 1:**
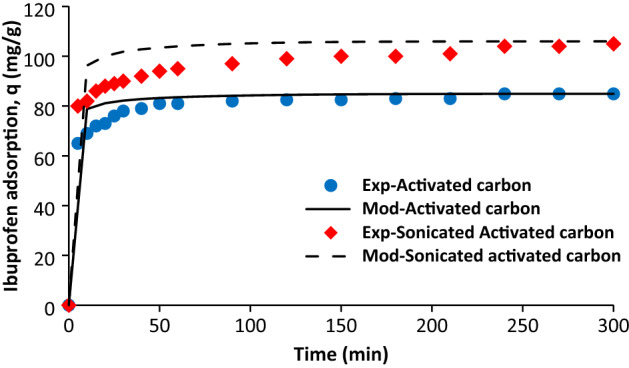
Comparison of experimental data^30^ with modelling values, initial ibuprofen concentration = 100 mg/L, T = 298 K, pH = 2, AC porosity = 0.392, SAC porosity = 0.474, adsorbent dosage of 0.5 g/L, and particle radius = 163 µm.

Figure 1 compares the experimental data obtained from literature^19,30^ and modelling values in terms of ibuprofen adsorption as a function of time for activated carbon and sonicated activated carbon. It was observed that there is reasonable agreement between experimental data and modelling results. Moreover, it was seen sharp increase of adsorption for both adsorbents at initial step, after that, the ibuprofen adsorption rate was decreased until reaching the equilibrium. The ibuprofen adsorption on sonicated AC was higher than the AC. It could be attributed to the higher porosity of SAC in comparison with AC (Table 1).

**Figure 2:**
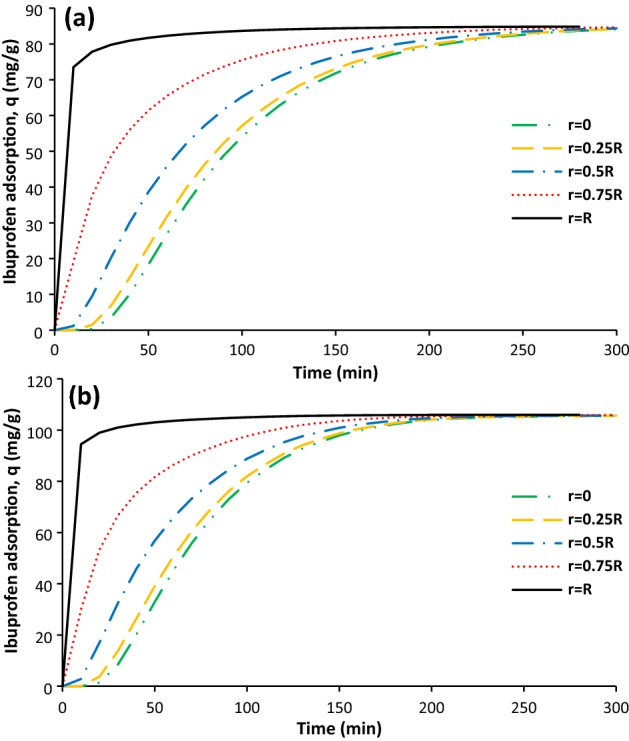
Ibuprofen adsorption as function of time at different position in particle for AC (**a**) and SAC (**b** initial ibuprofen concentration = 100 mg/L, T = 298 K, pH = 2, AC porosity = 0.392, SAC porosity = 0.474, adsorbent dosage of 0.5 g/L, and particle radius = 163 µm.

The ibuprofen adsorption profile as a function of time from centre of particle to its surface for both adsorbents was shown in Fig. 2a,b. As it can be seen, the needed time for reaching maximum adsorption increases for the positions close to the centre of particle. The ibuprofen adsorption was started at time of about 25 min for the activated carbon and about 10 min for the sonicated activated carbon at the centre of particle. This could be attributed to the particles porosity as the porosity of AC and SAC was 39% and 47% respectively. The higher porosity means the higher ibuprofen diffusion coefficient into the particle pores. The difference between ibuprofen adsorption profile is profound for the regions which have higher than r = 0.5R but it was observed slight difference between the adsorption curves of r = 0 and r = 0.25R for both AC and SAC.

**Figure 3:**
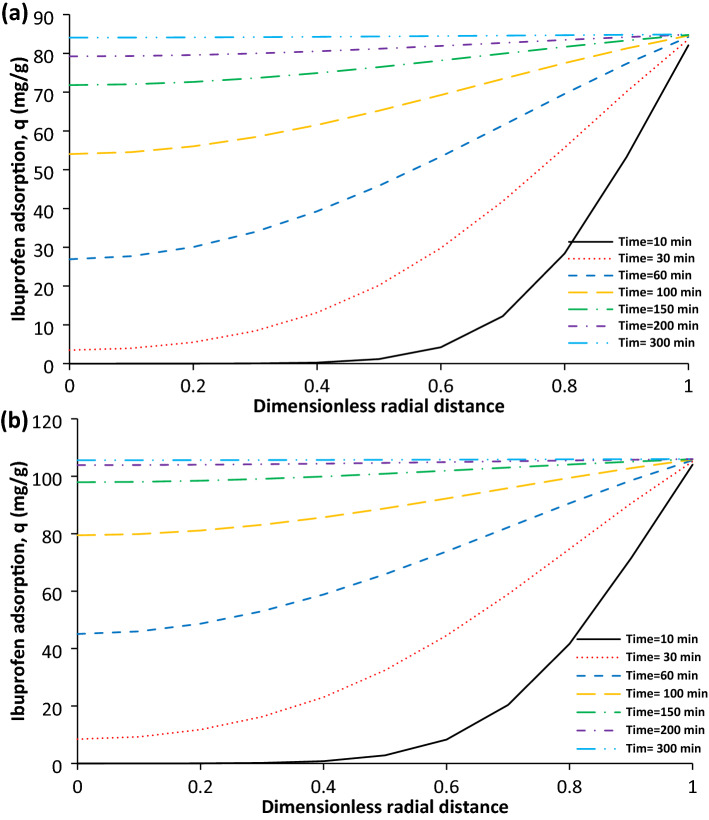
The ibuprofen adsorption as function of dimensionless radial distance at different times for AC (**a**) and SAC (**b**), initial ibuprofen concentration = 100 mg/L, T = 298 K, pH = 2, AC porosity = 0.392, SAC porosity = 0.474, adsorbent dosage of 0.5 g/L, and particle radius = 163 µm.

The ibuprofen adsorption as function of dimensionless radial distance at different times for AC and SAC was shown in Fig. 3a,b. It was not observed any ibuprofen adsorption within the zone of 0–0.42 and 0–0.36 at the first 10 min for AC and SAC respectively. At time of 150 min, the 84.5% and 92.5% of maximum adsorption was achieved for the AC and SAC at the centre of particle. As it can be seen, investigation of solute adsorption inside particle at different time point and positions can provide valuable information to understand the process accurately. As it can be seen, the solute adsorption at different zones of particle and time points can be investigated. In the literature, various kinetic models such as Langmuir–Hinshelwood used for only the prediction of the solute adsorption as function of time^33^.

### Effect of particle diameter

**Figure 4:**
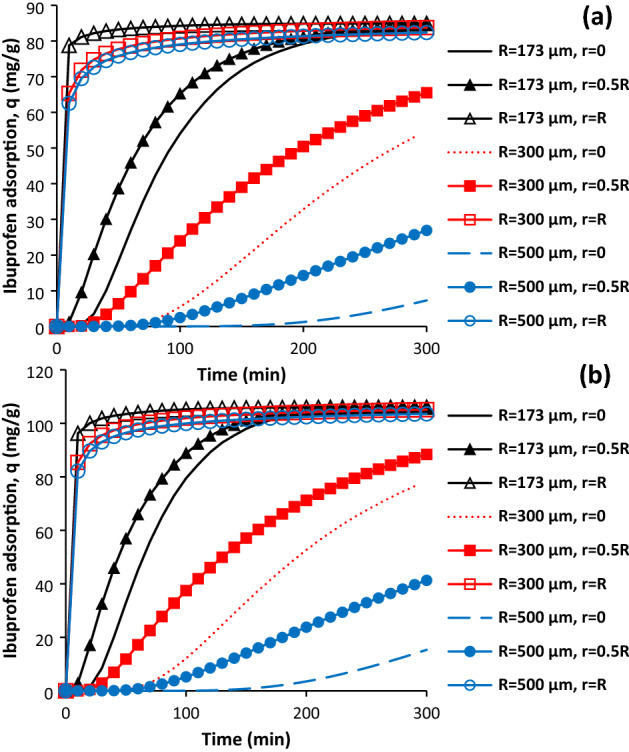
Effect of particle diameter on the ibuprofen adsorption for AC (**a**) and SAC (**b**), initial ibuprofen concentration = 100 mg/L, T = 298 K, pH = 2, AC porosity = 0.392, SAC porosity = 0.474, and adsorbent dosage of 0.5 g/L.

Figure 4a,b show the effect of radius of particle on the ibuprofen adsorption as function of time at different zones. According to Fig. 4a,b, increasing the particle diameter can have considerable influence of the ibuprofen adsorption and its distribution within the particle. The ibuprofen adsorption at the centre of particle was obtained after about 200 min and 150 min for AC and SAC when the radius was equal to 500 µm. Furthermore, the ibuprofen adsorption was reached to its maximum value for the particle with radius of 173 µm at centre of particle but more time is required to achieve the maximum adsorption for the particles with radius of 300 µm and 500 µm. In fact, the amount of adsorption is 8.60% and 14.5% of maximum adsorption for AC and SAC at the centre of particle (r = 500 µm) after 300 min. As it can be seen the particle with higher porosity has higher ibuprofen adsorption. In the middle of particle for both samples, there is firstly lag phase, then, the adsorption was started at high rate, then, it was gradually decreased with progressing of the adsorption time.

**Figure 5:**
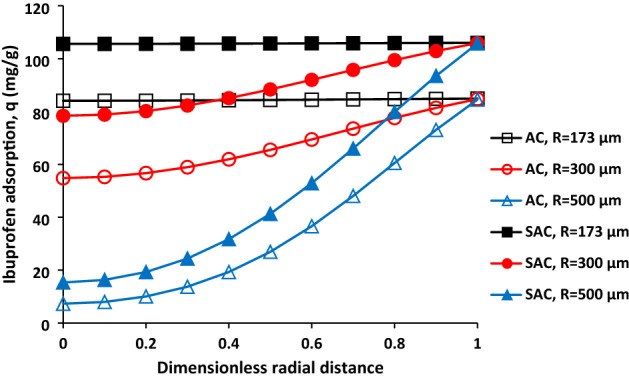
Ibuprofen adsorption as a function of dimensionless radial distance for AC and SAC with different particle diameters, initial ibuprofen concentration = 100 mg/L, T = 298 K, pH = 2, AC porosity = 0.392, SAC porosity = 0.474, and adsorbent dosage of 0.5 g/L.

Ibuprofen adsorption as function of radial distance at the end of adsorption process (time = 300 min) for AC and SAC was shown in Fig. 5. The ibuprofen adsorption is like horizontal line for the both sample with r = 173 µm. it means that there is no difference in terms of ibuprofen adsorption at centre and the particle surface. However, the ibuprofen adsorption was decreased from 84.10 to 54.81 mg/g and 7.30 mg/g with increasing the particle radius from 173 to 300 µm and 500 µm for the AC. In terms of SAC, the reduction in the ibuprofen adsorption was obtained 27.21 mg/g and 90.27 mg/g with the enhancement of particle diameter to 300 µm and 500 µm.

### Effect of particle porosity

**Figure 6:**
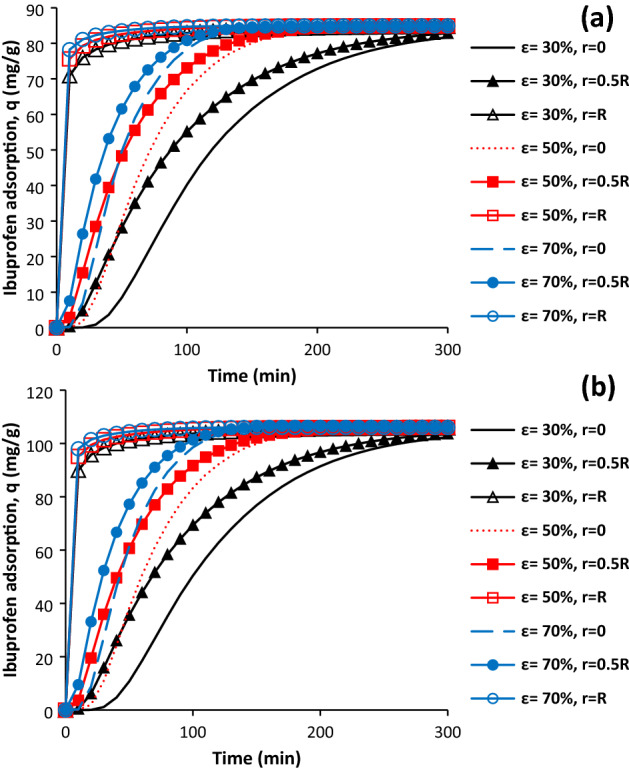
Effect of particle porosity on the ibuprofen adsorption for AC (**a**) and SAC (**b**), initial ibuprofen concentration = 100 mg/L, T = 298 K, pH = 2, adsorbent dosage of 0.5 g/L, and particle radius = 163 µm.

The particle porosity is an important parameter as it has influence on the ibuprofen diffusion coefficient within particle. Effect of particle porosity on the ibuprofen adsorption profile at different zones for AC and SAC was provided in Fig. 6. Generally, there is firstly sharp increase in ibuprofen adsorption, then, the rate of ibuprofen adsorption was decreased until end of process. Increase in porosity leads to the enhancement of solute diffusion coefficient within particle and decreasing of its density. Shorter time was required for reaching maximum adsorption when the porosity was higher. It should be pointed out that the maximum ibuprofen adsorption was not obtained for the particle with porosity of 30% at middle (r = 0.5R) and centre (r = 0) for both samples but it was close to the maximum value. For the particle with the porosity of 50% and 70%, 220 min and 140 min were required for obtaining maximum ibuprofen adsorption at the centre of particle.

**Figure 7:**
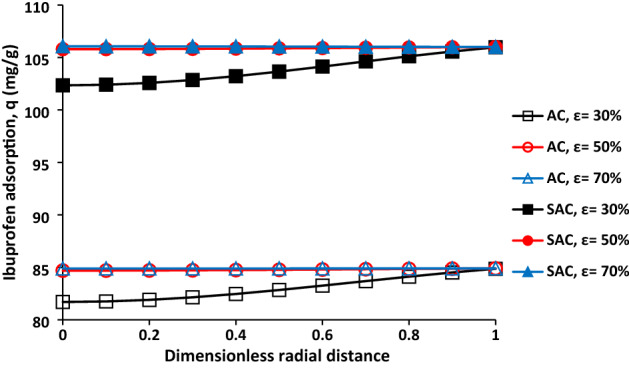
Ibuprofen adsorption as a function of dimensionless radial distance for AC (**a**) and SAC (**b**) with different particle porosity, initial ibuprofen concentration = 100 mg/L, T = 298 K, pH = 2, adsorbent dosage of 0.5 g/L, and particle radius = 163 µm.

Ibuprofen adsorption as a function of dimensionless radial distance for AC and SAC with different particle porosity was provided in Fig. 7. It was obtained at the end adsorption process (time = 300 min). It can be seen, the particles with porosities of 50% and 70% were reached the maximum adsorption in all area of particle. However, the ibuprofen adsorption was slightly decreased as the solute penetrates into deeper zones of the particle.

### Effect of solute diffusion coefficient in solution

**Figure 8:**
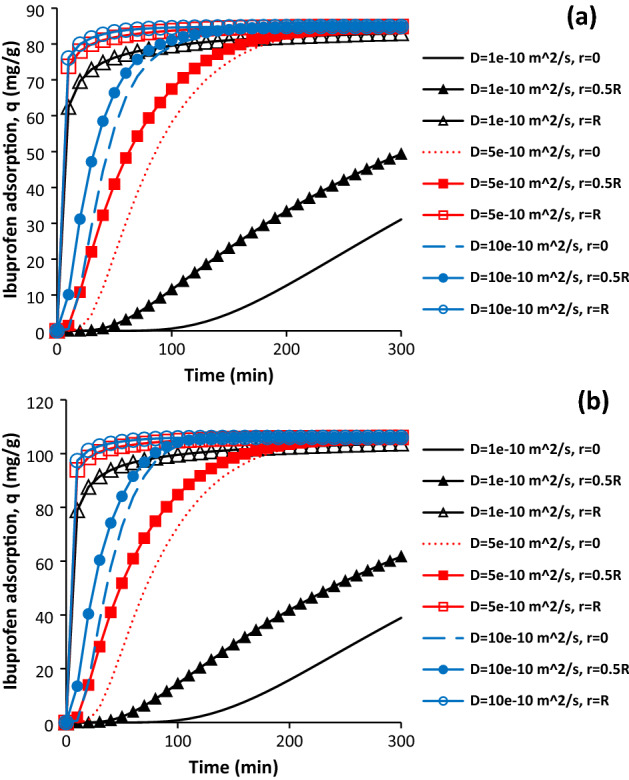
Effect of solute diffusion coefficient on the ibuprofen adsorption for AC (**a**) and SAC (**b**), initial ibuprofen concentration = 100 mg/L, T = 298 K, pH = 2, AC porosity = 0.392, SAC porosity = 0.474, adsorbent dosage of 0.5 g/L, and particle radius = 163 µm.

To investigate solute diffusion coefficient in solution on ibuprofen adsorption, it was changed from 1e^−10^ m^2^/s 10e^−10^ m^2^/s. Figure 8a,b show change in ibuprofen adsorption as a function of time with different ibuprofen diffusion coefficient at different distance from the particle centre for AC and SAC. In terms of activated carbon, the ibuprofen adsorption was observed after 50 min and 100 min at r = 0.5R and r = 0 when the diffusion coefficient was 1e^−10^. After 300 min, the adsorption was reached to 49.31 mg/g and 31.07 mg/g respectively. This behaviour means that more time is required for reaching maximum adsorption. Increasing solute diffusion coefficient resulted in the reduction of required time for reaching the maximum adsorption at different regions of the particle. In fact, higher diffusion coefficient facilitates ibuprofen adsorption into the adsorbent. The amount of adsorption was achieved more than 80 mg/g at the centre of particle after 160 min and 100 min when the solute diffusion coefficient was 5e^−10^ and 10e^−10^ m^2^/s, respectively. Sonicated activated carbon also had similar behaviour with change in solute diffusion coefficient in the solution. For the sample with 1e^−10^ m^2^/s, the amount of adsorption was reached 38.92 mg/g and 61.71 mg/g after 300 min at r = 0 andr = 0.5R. Therefore, it could be concluded that improving the solute diffusion coefficient for example by increasing the system temperature can increase the amount of adsorption rate into the adsorbent.

**Figure 9:**
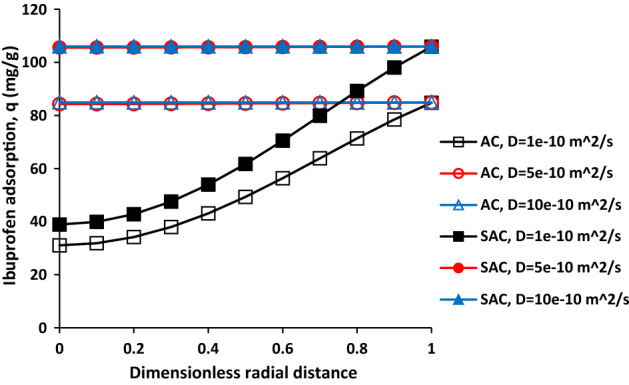
Ibuprofen adsorption as a function of dimensionless radial distance for AC and SAC with different solute diffusion coefficient, initial ibuprofen concentration = 100 mg/L, T = 298 K, pH = 2, AC porosity = 0.392, SAC porosity = 0.474, adsorbent dosage of 0.5 g/L, and particle radius = 163 µm.

The ibuprofen adsorption as function of dimensionless radial distance with different solute diffusion coefficient for AC and SAC was shown in Fig. 9. As it can be seen, there is no difference in ibuprofen adsorption in the range of the particle centre to its surface at the end of adsorption process when the solute diffusion coefficient is 5e^−10^ and 1e^−9^ m^2^/s. However, it can be seen decrease in the ibuprofen adsorption towards the particle centre when the solute diffusion coefficient is 1e^−10^ m^2^/s for both samples. The ibuprofen adsorption was increased from 31. 07 mg/g and 38.92 mg/g to 84.78 mg/g and 105.92 mg/g with change in the position from r = 0 to r = R for AC and SAC respectively. Lower solute diffusion coefficient needs more time to have the same adsorption value in all regions of particle.

## Conclusion

The adsorption of ibuprofen onto activated carbon and sonicated activated carbon was theoretically investigated in this study. The effect of the model parameters on the shape of the ibuprofen adsorption profile as function of time or dimensionless radial distance was discussed in detail. The modelling values were successfully validated using experimental data reported in literature. Change in the particle characteristics and solute diffusion coefficient had significant influence on ibuprofen adsorption profile especially at the centre of particle. The SAC showed better performance than AC as it had higher porosity and external mass transfer coefficient. Higher particle diameter and lower particle porosity and solute diffusion coefficient was required higher time in order to reach the maximum ibuprofen adsorption in particular at the centre of particle.
